# Cellular Therapies in Chronic Granulomatous Disease

**DOI:** 10.3389/fped.2020.00327

**Published:** 2020-06-26

**Authors:** Tayfun Güngör, Robert Chiesa

**Affiliations:** ^1^Department of Immunology, Hematology, Oncology and Stem Cell Transplantation, University Children's Hospital Zürich, Zurich, Switzerland; ^2^Department of Bone Marrow Transplantation, Great Ormond Street Hospital for Sick Children, London, United Kingdom

**Keywords:** chronic granulomatous disease, CGD, hematopoietic stem cell transplantation, conditioning, therapeutic drug monitoring, serotherapy, gene therapy

## Abstract

Allogeneic hematopoietic stem cell transplantation (HSCT) has become the main curative treatment in patients with chronic granulomatous disease (CGD). CGD is caused by inherited defects of the phagolysomal NADPH-oxidase, leading to a lifelong propensity for invasive infections and granulomatous inflammation. After successful allogeneic HSCT, chronic infections and inflammation resolve and quality-of-life improves. Favorable long-term outcome after HSCT is dependent on the prevention of primary and secondary graft failure (GF), including falling myeloid donor chimerism (DC) below 10 %, and chronic graft-vs.-host-disease (cGVHD). The risk of GF and GvHD increases with the use of HLA-incompatible donors and this may outweigh the benefits of HSCT, mainly in patients with severe co-morbidities and in asymptomatic patients with residual NADPH-oxidase function. Seventeen scientific papers have reported on a total of 386 CGD-patients treated by HSCT with HLA-matched family/sibling (MFD/MSD), 9/10-/10/10-matched-unrelated volunteer (MUD) and cord blood donors. The median OS/EFS-rate of these 17 studies was 91 and 82%, respectively. The median rates of GF, cGVHD and *de-novo* autoimmune diseases were 14, 10, and 12%, respectively. Results after MFD/MSD and 10/10-MUD-transplants were rather similar, but outcome in adults with significant co-morbidities and after transplants with 9/10 HLA-MUD were less successful, mainly due to increased GF and chronic GVHD. Transplantation protocols using T-cell depleted haploidentical donors with post-transplant cyclophosphamide or TCR-alpha/beta depletion have recently reported promising results. Autologous gene-therapy after lentiviral transduction of HSC achieved OS/EFS-rates of 78/67%, respectively. Careful retrospective and prospective studies are mandatory to ascertain the most effective cellular therapies in patients with CGD.

## Introduction

Chronic granulomatous disease is caused by mutations leading to defects in individual subunits of the phagocyte NADPH-oxidase (gp91phox in X-linked-; p22-, p47-, p67-, p40phox, and EROS in autosomal recessive-CGD) (1–4). The NADPH-oxidase-myeloperoxidase system generates microbicidal oxidants required for host defense and control of inflammation. CGD affects ~1:200,000–250,000 live-births (5–7) and X-linked-CGD accounts for approximately two-thirds of patients. P47phox-mutations are the most common AR-defects. Rarely, female carriers of X-CGD with random X-lyonization of <15% of circulating NADPH-oxidase-producing neutrophils present with CGD-symptoms (7–9). Symptoms comprise of invasive infections and chronic autoinflammatory diseases leading to frequent medical interventions, impaired quality-of-life, and increased morbidity/mortality (10–13). The majority of patients are diagnosed in childhood, while some develop symptoms in adulthood (7, 14, 15). Due to residual NADPH-oxidase activity, patients with AR-p47phox-mutations survive longer than X-CGD-patients (survival >40 years: >80 vs. 55%) (16). The clinical course may be unpredictable even in individuals of families with identical CGD-mutations (17). Short stature, osteoporosis, organ failure, and amyloidosis are long-term complications (18). There is still paucity of data on quality-of-life and emotional health in patients with CGD (11, 12, 14, 19, 20). Today, 90% of children with CGD are reaching adulthood and the transition into adult care is challenging (21, 22).

The infections typically affect lungs, lymph nodes, skin, liver, perianal region, gingiva and bone and are mainly caused by Staphylococcus aureus, Burkholderia cepacia, Nocardia, Serratia marcescens, and Aspergillus species. Klebsiella pneumoniae, Salmonella (7, 23), Mycobacteria (21, 24), Actinomyces, Granulibacter bethesthensis (25–27). Infections caused by Chromobacterium violaceum and B. pseudomallei (28–30) are less frequently encountered. The use of life-long antibacterial prophylaxis with trimethoprim-sulfamethoxazole is recommended. Pulmonary Aspergillus-infections are the leading cause of mortality (31). Anti-fungal prophylaxis, mainly with itraconazole (32, 33), can reduce the incidence of fungal infections, but the emergence of azole-resistant aspergillus species and dematiaceous molds is becoming a clinical challenge (34).

Absent or reduced NADPH-oxidase activity in monocytes/macrophages causes impairment of efferocytosis and autophagy (35, 36). Ineffective apoptotic cell clearance increases the risks of developing autoinflammation (37). Progressive granulomatous lung disease (PGLD), Crohn-like enterocolitis (38, 39) and obstructive genitourinary inflammation (40, 41) are relevant autoinflammatory syndromes and their risk increase steadily during life (14). Initial gastrointestinal involvement without infections has often been misdiagnosed as Crohn‘s disease (38, 39). Treatment of autoinflammation includes steroids (42) and more recently IL1- or TNF-alpha inhibitors to replace steroids, however, all of these drugs bear the risk of increasing the risks for invasive infections (43). Thalidomide (44–46), vedolizumab, ustekinumab (47, 48), as well as pioglitazone (36) can be beneficial to reduce autoinflammation in CGD and regular IFN-gamma injections decrease the incidence of bacterial infections with no impact on the incidence of colitis (21, 49–55).

Before HSCT, screening for infections is mandatory in biopsies of infectious lesions and in bronchoalveolar lavage specimens (14, 56). Steroids added to antimicrobials can accelerate the regression of infectious lesions (42, 57, 58) and can help to avoid extensive surgery (59). Granulocyte-transfusions should be strictly indicated to prevent CMV-transmission and sensitization to blood cell antigens (60–62). The McLeod-blood group should be evaluated in X-CGD-patients to minimize the sensitization against Kx-positive red cell transfusions (63–65).

## HSCT MAC-Conditioning (Table 1)

In Europe, the first major survey of the Inborn Errors Working Party of the EBMT reported on 27 patients with CGD who had been transplanted between 1985 and 2000. At HSCT, nine of 27 patients had intractable invasive infections and received antibiotics as well as granulocyte-transfusions (seven of nine). Eighteen of 27 patients were free of infection at HSCT. Seven of the 18 patients without overt infection had signs of active ongoing autoinflammation including enterocolitis and PGLD. Twenty-five of the 27 patients received MSD-transplants (five heterozygous carriers). Two patients with no overt infection or autoinflammation received a MUD-transplant. Conditioning-regimens were mainly myeloablative with full-dose busulfan/cyclophosphamide and mainly without serotherapy (67). Recovery from refractory infection, remission of inflammatory organ dysfunction and catch-up growth were observed (67). Patients without overt autoinflammation/infections had an OS of 100%, whereas patients with ongoing infections at transplant had a TRM of 44% (four of nine) (67). The OS/EFS was 85/81%, respectively. The GF and chronic GVHD rate were 7 and 11%, respectively. The majority of surviving patients had >95% circulating myeloid cells of donor origin. This important paper showed that myeloablative HSCT based on busulfan/cyclophosphamide and no *in vivo* T-cell depletion was overall efficient in sibling transplants but induced exuberant inflammation in patients suffering from ongoing infections at transplant. The same was observed in a transplantation model in non-infected CGD mice after myeloablative allogeneic HSCT resulting in marked infiltration of the lungs with inflammatory cells, in contrast to normal mice (81). Cultured monocytes from the CGD-mice produced 3-fold TNF-alpha (81), explaining the higher incidence of severe GvHD in patients with pre-existing overt infections treated with HSCT without serotherapy. Myeloablative regimens containing cyclophosphamide were greatly abandoned in Europe after this experience. The authors at that time concluded that all infectious/inflammatory foci had to be detected and treated before HSCT and that HSCT should be mainly restricted to children with MSD/MFD (67).

**Table 1:** Major HSCT studies with HLA-matched donor transplants in CGD between 2001 and 2019 (*n* >5 patients).

Author^*^Year of report	**Horwitz et al. (66)**	**Seger et al. (67)**	**Schuetz et al. (68)**	**Soncini et al. (69)**	**Gozdzik et al. (70)**	Martinez^**^ et al. (71)	**Tewari et al. (72)**	**Ahlin et al. (73)**	**Gungor et al. (63)**	**Morillo-Gutierrezet al. (74)**	**Khandelwal et al. (75)**	**Parta et al. (64)**	**Osumi et al. (76)**	Yanir^**^ et al. (77)	**Fox et al. (78)**	**Arnold et al. (79)**	**Lum et al. (80)**
**Conditioning**	RIC Flu/CY + rATG	MAC (most) Bu/CY ±TNI/TT /Mel /rATG	MAC (most) Bu/Cy ± TBI, Mel, RIT ± rATG	MAC (most) Bu/Cy +Camp.	MAC (most) Bu/Cy or Flu/Mel + rATG	MAC Bu/Cy + Flu + Campath	MAC Bu/Cy + Flu + −2 Gy TBI + eATG	RIC and RTC/MAC1. Bu/CY2. Bu/Flu 3. Treo/Flu ± rATG	RIC Flu/low Bu + rATG or Campath	RTC Flu/Treo/ or Cy or TBI ± rabbit ATG or Camp.	RIC and RTC/MAC Flu/Mel + Camp. Or Bu/CY + rATG	RTC Bu (10 mg/kg)/TBI 3 Gy, +Camp.	RTC Flu/Bu/TBI 3 Gy + ATG	MAC Bu/Flu/CY + Ara C, + Camp.	**RIC** 1. Flu/Bu +Camp. or rATG (1) 2. Flu/Mel +Camp. (2)	MAC Bu/Flu +ATG (+TT)	MAC RIC/RTC Mixed
TDM	No	No	No	No	No	No	No	No	Yes	No	Yes	No	Yes	Yes	Yes	Yes	No
Target Bu cAUC Achieved cAUC mg /Lx h	No	No	No	No	No	No	No	No	Yes 45–65 30–65	No	Yes 60–70 59–66	No 19–88	Yes 45–65 39–52	No 60–80	No 44–63	No 59–98	No
Ped/Adult Age (yrs)	Mixed 5–36	Mixed 3–39	Mixed 4–20	Mainly Ped. 1–21	Ped. 2–13	Ped 1–13	Ped. 0.7–11.7	Mixed 1–35	Mixed 1–39	Ped. 0.4–19	Mainly Ped. 0.45–20.17	Mixed 4–32	Ped. 2–18	Mixed 0.5–30	Adult 17–28	Ped. 1–13	Ped. 0.6–18
**Patients** ***n*****=**	10	27	12	20	6	11	12	14	56	70	18	40	6	24 (11^**^)	11	7	55
X-linked CGD %	80	85	92	95	83	82	67	71	61	80	44	85	83	88	64	86	82
Colitis%	ND	7	0	50	0	9	30	14	34	50	11	12.5 (?)	67	0	55	43	91
PGLD%	ND	26	58	25	67	27	ND	ND	14	21	11	0	0	4	0	0	7
Lung infection %	ND	26	83	25	33	55	17	43	27	17	0	20	0	25	27	14	?
Liver abscess %	ND	0	40	15	0	9	8	7	2	0	0	2.5	0	0	0	14	?
McLeod *n*=	ND	ND	2	ND	ND	ND	ND	2	1	1	0	2	0	0	1	0	?
**Donor** ***n*****=**																	
MSD/MFD *n*= (carrier *n*=)	10	25 (5)	3	10 (1)	2	4	6	5 (2)	21	13	3	6	0	6	3	1	20
MUD 10/10	0	2	9	10	3	7	0	7	25	44	15	33	3	16	6 (10/10 MUD)	7	31
MUD 9/10	0	0	0	0	0	0	0	0	10	11	0	1	3	4	2 (9/10 MUD)	0	ND
MMUD 8/10	0	0	0	0	1	0	0	0	0	1 haplo	0	0	0	0	8	0	4 haplo
UCB 4-6/6	0	0	0	0	0	0	8	4	0	1	0	0	0	0	2	0	2
Source *n*=	0 BM	24 BM	9 BM	15 BM	6 BM	11	5 BM	11 BM	45 BM	36 BM	16 BM	5 BM	6 BM	ND BM	4 PBSC	6 BM	53 BM
	10 PBSC	3 PBSC	3 PBSC	3 PBSC	0 PBSC	0 PBSC	0 PBSC	3 PBSC	11 PBSC	33 PBSC	1 PBSC	35 PBSC	0 PBSC	ND PBSC	7 BM	2 PBSC	23 PBSC
	0 CB	0 CB	0 CB	2 CB	0 CB	0 CB	9 CB	4 CB	0 CB	1 CB	1 CB	0 CB	0 CB	0 CB	0 CB	0 CB	2 CB
**Outcome**																	
Med. FU in months	17	24	53	61	20	48	70.5	92	21	34	20 (in RIC) 60 (in RTC/MAC)	41	12	48	33	32	78
OS %	70	85	75	90	100	100	100	93	96	90.5	83	82.5	100	91	81.8	100	89
EFS %	60	81	67	90	83	100	100	79	91	81	50	80	83	83	90.9	90	77
Myeloid DC% *n* = pat.	6 (full) 2 (mix.)	22 (full)	11 (full)	14 (full)	100%	9 (full) 2 (mix.)	15 (>90%)	12 (>90%) 1 (60%)	52 (>90%)	51 (>95%) 1 (<90%) 3 (39–74%)	14 (>95%) 1 (50%) 2 (11–40%)	27 (>97%) 3 (>70%) 1 (<50%)	5 (>95%) 1 (0%)	22 (full) 2 (mixed)	4 (100 %) 7 (mixed)	7 (full)	43 (Med. 92%)
DLI/SCB	9/0	2/0	0/0	0/0	1/0	0/0	0/0	3/0	0/0	4/0	1/2	0/6	0/0	0/0	0/0	0/0	0/3
Re-HSCT	0	0	1	1	1	0	3	3	3	5	0	3	1	2	0	1	4
DSF after re-HSCT%	NA	NA	0	100	100	NA	100	67	67	80	NA	0	ND	100	NA	100	100
Graft failure % (*n*=)	20 (2)	7 (2)	17 (2)	5 (1)	16 (1)	0 (0)	25 (3)	14 (2)	5 (3)	12 (8)	50 (2)	22 (9)	17 (1)	8 (2)	0	15 (1)	7 (4)
aGVHD III-IV % (*n*=)	10 (1)	15 (4)	0 (0)	10 (2)	17 (1)	0 (0)	8 (1)	7 (1)	4 (2)	12 (8)	28% (5)	15% (6)	17% (1)	0% (0)	1	15 (1)	9 (5)
chronic GVHD % (*n*=)	20 (2)	11 (3)	8 (1)	10 (2)	17 (1)	0 (0)	33 (4)	0 (0)	7 (4)	13 (9)	22 (4)	12.5 (5)	17 (1)	0 (0)	3 (1)	0 (0)	0 (0)
Lethal infections (*n*=)	Bact. (1) Fung. (1)	Fung. (2) Pre-exist.	BK (1) CNS (1) ARDS (1)	Fung. (2) Pre-exist.	(0)	(0)	(0)	Fung. (1) Pre-exist.	PTLD (1)	Bact. (1), ADV (2), FLU (1)	Fung. (1)	Bact. (2), Fung. (1) Pre-exist.	Not specified (2)	(0)	ND	(0)	PTLD (1)
*De-novo* autoimmunity % Type (n=)	ND	ND	ND	11 Thyroid (2)	ND	18 Thyroid (1) AIHA (1)	25 ITP (3)	7 AIN (1)	4 AIHA (2)	5 AIHA (2) GBS (1)	ND	2.5 AIHA/ITP (1)	ND	50 AIHA/ITP (6) Thyroid (6) GB (2)	ND	14 AIHA (1)	12 AIHA (3) Thyroid (2) DM (1)
Reported fertility (*n*=)	ND	ND	ND	ND	ND	ND	ND	ND	Fatherhood. (2)	ND	ND	ND	ND	ND	Fatherh. (1) Viable sperm (2)	ND	ND

*ADV, adenovirus; aGVHD, acute Graft-vs.-host disease; AIHA, autoimmune hemolytic anemia; AIN, autoimmune neutropenia; ARDS, acute respiratory distress syndrome; Bact, bacteria; Bu, Busulfan; Camp, Campath IH/Alemtuzumab; chronic GVHD, chronic graft-vs.-host disease; CY, cyclophosphamide; DC, donor chimerism; DFS, disease-free survival; DLI, donor lymphocyte infusion; DM, diabetes mellitus, eATG, equine Anti T-cell globulin; EFS, event free survival; Fatherh., fatherhood; Flu, fludarabine; FU, follow-up; Fung, fungi; GB, Guillain-Barré-Syndrome; ICH, intracranial hemorrhage; ITP, immune thrombocytopenia; LD, lung disease; MAC, myeloablative conditioning; med, median; Mel, melphalan; MSD, matched family donor; MFD, matched family donor; MMUD, mismatched unrelated donor; MUD, matched unrelated donor; NA, not applicable; ND, not done; OS, overall survival; PGLD, progressive granulomatous lung disease; SCB, stem cell boost; TDM, therapeutic drug monitoring; rATG, rabbit Anti T-cell/thymocyte globulin; RIC, reduced intensity conditioning; RTC, reduced toxicity conditioning; RIT, radioimmune therapy; SCB, stem cell boost; TDM, therapeutic drug monitoring; TBI, total body irradiation; TNI, total nodal irradiation; Treo, treosulfan; TT, thiotepa; UCB, unrelated cord blood*.

* Literature citation see in main manuscript.

** *11 patients from Martinez were reanalyzed with a longer follow-up in Yanir‘s investigation*.

## HSCT With RIC/RTC-Regimens (Table 1)

Almost simultaneously to the above mentioned European experience, the NIH in the USA used for the first time a reduced intensity conditioning (RIC) comprising of non-myeloablative fludarabine/cyclophosphamide followed by *in-vitro* T-cell depleted grafts. This approach resulted in clearly increased GF-rates (20%), even with the use of matched family/sibling donors (66). Donor-lymphocyte infusions were necessary to prevent falling DC but unfortunately induced severe acute GVHD and resulted in a transplant-related mortality rate of 30% (66, 82). RIC-regimens including melphalan and fludarabine were associated with similarly high GF-rates (30%) (75).

RIC-regimens based on reduced or targeted busulfan, fludarabine and serotherapy were more successful and achieved sufficient myeloablation and clearly lower rates of GF and chronic GVHD (38, 63, 83–85). These busulfan-fludarabine-based RIC-regimens were first used in adult high-risk CGD-patients suffering from invasive Aspergillus-infections and/or enterocolitis using MSD/MFD- or MUD transplants. The OS/EFS rates were 100% in these small initial series (38, 84). Administration of anti-T-cell/thymocyte globulins as well as of a humanized monoclonal anti-CD52 antibody (Campath IH; alemtuzumab) were shown to deplete successfully T-cells and allo-stimulatory dendritic cells (86) of recipient origin. The importance of using serotherapy for *in-vivo* T-cell depletion to reduce both GF and chronic GVHD after HSCT for CGD became obvious. Viral reactivations after serotherapy were fortunately rare or well manageable rendering clinical HSCT outcomes with MUD-donors vastly similar to MSD/MFD-donors (68, 69, 71).

Busulfan-based RIC-conditioning was further refined by investigating the interindividually variable busulfan clearance and exposure in patients (87, 88). Therapeutic drug monitoring (TDM) helped optimize both safety and efficacy of busulfan-administration. The assessment of the cumulative AUC (cAUC) turned out as an appropriate tool to measure the total busulfan-exposure and -toxicity (87, 89). A 10-year prospective study on 56 pediatric/adult CGD-patients (2/3 high-risk patients) treated with submyeloablative busulfan (half-dose or cAUC 45–65 mg/L × h) yielded, indeed, excellent results. Busulfan-dose adjustments (90) were necessary in 14/44 patients (32%) (63). Immunoablation was achieved with fludarabine and serotherapy including rabbit ATG or alemtuzumab. After a follow-up time of 21 months, the OS/EFS-rates were 93 and 89%, respectively. However, GF could not be abolished and occurred in 5% of patients. The cumulative incidences of grade III–IV acute GVHD and chronic GHVD were low with 4 and 7%, respectively. Stable ≥90% myeloid DC was documented in 93% surviving patients leading to resolution of infectious and inflammatory lesions. Equivalent outcomes were observed between MFD/MSD and MUD rendering matched unrelated donors a good donor choice in the absence of matched sibling donors. Outcomes were not different between 9/10-HLA- (*n* = 10) and 10/10-matched MUD (*n* = 25), but the numbers were low. Two fatherhoods were documented after successful HSCT. To further reduce the risk of graft failure with this RIC-regimen, some investigators have narrowed the submyeloablative target of the cumulative AUC of busulfan to 55–65 mg/L × h (83) and have started using busulfan starting doses based on a new body weight-dependent busulfan dosing nomogram (91).

Morillo-Gutierrez et al. (74) showed in a large retrospective European study of the EBMT on 70 CGD-children that HSCT after treosulfan-based conditioning was well tolerated and achieved OS/EFS-rates of 91.4/81.4%, respectively. Treosulfan, an alkylating drug with both myeloablative and immunosuppressive effects, exhibited an overall low acute toxicity in CGD transplants. If used as a single alkylator, treosulfan may be less gonadotoxic than other alkylators, however, there is no study yet available convincingly proving this assumption (92–94). Excellent myeloid DC (>95%) was documented in 80% of surviving patients. With this paper, treosulfan-based RTC was shown to be an alternative conditioning to targeted busulfan-based-RIC, although it remained unclear which treosulfan systemic exposure was more likely to be myeloablative or submyeloablative. Graft failure remained a problem occurring in 12% of the patients (74). Some centers have therefore started to add thiotepa to treosulfan to further reduce the risk of GF (80), probably at the expense of augmented gonadotoxicity (94).

The experience with unrelated 4/6–6/6-HLA-compatible cord blood transplants (CBT) in CGD is scarce, but there are a few examples of successful transplants using cord blood in patients lacking MSD or MUD (72, 95, 96). Due to low HSC-numbers in CB, CBT is usually restricted to patients with low body weight (<20 kg) and viral reactivations may be of concern. CBT usually requires myeloablative and therefore more gonadotoxic conditioning, e.g., busulfan (cAUC 80–100 mg/L × h) or treosulfan/thiotepa, to achieve sufficient myeloid engraftment.

For this review, we have analyzed the results of the two above mentioned major European studies together with 15 other relevant international papers published between 2010 and 2019. We have summarized the results of 386 CGD-patients receiving transplants from mainly MSD/MFD- and MUD-donors in Table 1. The median overall incidences of OS, EFS, graft failure, chronic GVHD and *de-novo* autoimmune disease in these 17 papers were 92, 81, 14, 9, and 15%, respectively. The most important secondary problems were graft failure including patients with slowly falling myeloid DC <10% (DHR/NBT-tests <10%), *de-novo* autoimmunity and chronic GVHD. Graft failure or low donor myeloid DC was associated with reappearance of CGD associated symptoms, and chronic GVHD clearly impacted negatively on quality-of-life and life expectancy (Table 1).

## HSCT With Haploidentical Donors and Gene-Therapy (Table 2)

Hoenig et al. demonstrated for the first time that haploidentical HSCT was curative CGD (97). They used myeloablative conditioning (full-dosed busulfan, thiotepa and alemtuzumab) and *in-vitro* selected peripheral HSCs and achieved full donor donor cell engraftment and complete resolution of pulmonary aspergillosis. More recently, haploidentical TCR alpha-beta -/CD19-depleted grafts were shown to successfully achieve myeloid donor cell engraftment without inducing relevant GVHD (103–105). The advantage of these *in-vitro* T-cell depletion techniques is that chronic GVHD is rare (14, 80, 106). *In-vivo* T-cell depletion strategies in haploidentical transplants include the use post-transplant cyclophosphamide (PT/CY) (50 mg/kg/day), administered on day+3 and day +4 (107). PT/CY is non-toxic to donor HSCs, but efficiently eliminates activated alloreactive donor-derived CD3+T-cells while sparing resting CD3+T-cells with potential anti-infective properties. The first successful haplo HSCT with PT/CY in CGD was reported in the USA after the administration of targeted busulfan (cAUC 40 mg/L × h), fludarabine, cyclophosphamide and 2 Gy TBI (98). However, in a very recent follow-up paper by Parta et al. on seven patients with CGD a rather high rate of severe GVHD was observed leading to death in two patients (OS and EFS 71%, respectively). The estimated total cumulative of busulfan ranged from 30–52 mg/L × h (2,461–4,250 min × micromol/L × 3 days). They used a protocol with mainly PBSC grafts and sirolimus for GVHD-prophylaxis (101). Patients' age ranged between 14 and 26 years and comprised of mainly adults. Severe grade III acute GVHD were observed in three patients with enterocolitis.

**Table 2:** Haploidentical HSCT and autologous gene-therapy in CGD between 2014 and 2020.

Author^*^Year of report	**Hoenig et al. (97)**	**Parta et al. (98)**	Shah et al.^**^ (99)	**Reguiero-Garcia et al. (100)**	Lum et al. ^**^ (80)	**Parta et al. (101)**	**Kohn et al. (102)**
**Conditioning**	**MAC**	**RTC**	**RTC/MAC**	**RTC/MAC**	**RTC/MAC**	**RTC**	**Gene therapy**
(type of T-cell depletion)	Bu/Flu/TT CD34+ positive selection (*in-vitro*)	Bu/Flu/CY/TBI 2 Gy+post CY2 × 50 (d+3/+4) (*in-vivo*)	Treo/TT/Flu TCR-alpha/beta+/CD19+-depletion (*in-vitro*)	Treo-based +post CY 2 × 50 (d + 3/4) (*in-vivo*)	Treo/TT/Flu TCR-alpha/beta+/CD19+-depletion (*in-vitro*)	Bu/Flu/CY/TBI 2 Gy+post CY2 × 50 (d + 3/+4) (*in-vivo*)	Myeloablative Busulfan
TDM	No		No	No	No	Yes	Yes
Target Bu (total dose mg/kg) Achieved Cauc mg/L × h	No (17.6)	No (10.4) 37	No	No	No	No (10.4) 30–52	Target 70–75
Ped/Adult Age (yrs)	Ped (6)	Ped (14)	Ped (3)	Ped (ND)	ND ND	Mixed (14–26)	Mixed (2–27)
**Patients** ***n*****=**	**1**	**1**	**2**	**1**	**4**	**7**	**9**
X-linked CGD %	100	100	50	100	ND	71	100
Colitis %	0	100	50	ND	ND	86	11
PGLD %	0	0	0	ND	ND	0	22
Lung infection %	100	100	50	ND	ND	43	33
Liver abscess %	0	0	0	ND	ND	0	11
McLeod %	0	9	0	ND	ND	ND	0
**Donor**							
Type of transplant	1 haplo	1 haplo	2 haplo	1 haplo	4 haplo	7 haplo	9 autologous
Father	1	1	2	ND	ND	5 (1 brother)	NA
Mother	0	0	0	ND	ND	1 (10/10 phenoident.)	
Source	PBSC	PBSC	PBSC	ND	PBSC	PBSC	PBSC
**Outcome**							
Med. FU (mo.)	48	9	47	?	ND	36	ND (12–36)
OS%	100	100	100	100	100	71	78
EFS%	100	100	100	100	100	71	66
Myeloid DC% (*n*=)	90 (1)	100 (1)	100 (2)	ND	100 (4)	96–100 (7)	12–46 (7)^***^
DLI/SCB	0	0	0	ND	0	0	NA
Re-HSCT	0	0	0	ND	0	0	ND
Graft failure %	0	0	0	ND	0	0	22
Acute GVHD III-IV %	0	0	0	ND	0	43	NA
Chronic GvHD %	0	0	0	ND	0	29	NA
Lethal infections (*n*=)	0	0	0	0	0	2	Pneumonitis (1) ICH (1)
*De-novo* autoimmunity % Type (*n*=)	ND	ND	ND	ND	ND	ND	ND
Reported fertility (*n*=)	ND	ND	ND	ND	ND	ND	ND

Bu, Busulfan; cAUC, cumulative area under the curve; CY, cyclophosphamide; DC, donor chimerism; DLI, donor lymphocyte infusion; EFS, event free survival; Flu, fludarabine; FU, follow-up; GVHD, chronic graft-versus-host disease; haplo, haploidentical family donor; ICH, intracranial hemorrhage; PGLD, progressive granulomatous lung disease; MAC, myeloablative conditioning; NA, not applicable; ND, not determined; OS, overall survival; PGLD, progressive granulomatous lung disease; rATG, rabbit Anti T-cell globulin; RIC, reduced intensity conditioning; RTC, reduced toxicity conditioning; SCB, stem cell boost; TDM, therapeutic drug monitoring; Treo, treosulfan; TT, thiotepa.

* *Literature citation see main manuscript*.

** *Presumably patients of the same UK cohort*.

*** *Percentage of functional neutrophils*.

Another currently investigated RTC-protocol is currently used in our institution. It comprises of up-front rabbit ATG (30–40 mg/kg), fludarabine (180 mg/sqm) and targeted busulfan (cAUC 65–75 mg/L × h) followed by haplo-HSCT with PT/CY and GVHD prophylaxis with CSA and MMF (starting at day +5). We believe that both haploidentical HSCT with PT/CY and with antibodies containing magnetic beads are promising alternatives in high-risk patients with CGD when HLA-matched related or unrelated donors are unavailable (105). To further explore the rates of graft failure and cGVHD after haploidentical HSCT comparative studies of both techniques are urgently needed (Table 2).

Autologous gene-therapy (GT) of HSCs leads to partially functional correction of defective phagocytes and is a potentially curative treatment approach in CGD. Graft failure may occur after GT, but the risk of GVHD is zero (102, 108, 109). While early studies with unconditioned transfusions of retrovirally transduced HSCs were unsuccessful (110), autologous infusion of HSCs transduced with a gamma-retroviral vector after busulfan-based myeloablative conditioning helped to successfully engraft 4 CGD-patients (2 adults, 2 children) (109). Approximately 15% of gp91phox-expressing neutrophils had been detectable within the first 5 months after GT leading to resolution of life-threatening invasive fungal infections. Unfortunately, methylation with downregulation of the transduced gene and clonal expansion of transduced myeloid cells due to random viral integrations were observed, leading to activation of endogenous oncogenes and development of MDS with or without monosomy 7. Both children treated with GT survived after subsequent allogeneic HSCT (65), while 2 adult patients died due to secondary MDS and AML, respectively. Recently, nine X-CGD-patients (age 2–27 years) received GT using a self-inactivating lentiviral vector designed to limit the risk of mutagenesis (102). Patients were pretreated with myeloablative busulfan exposures (cAUC 70–75 mg/L × h). Two patients died within 3 months from GT due to severe pulmonary disease and hemorrhage. At 12 months, 6/7 surviving patients demonstrated persistence of sufficiently NADPH-oxidase-expressing neutrophils (16–46%) and stable vector copy numbers. One patient had graft failure with a decline <5% enzyme-producing neutrophils. There was no evidence of clonal dysregulation or transgene silencing. Surviving patients did not develop new CGD-related infections, and six have been able to discontinue antibiotic prophylaxis (OS/EFS >12 mo.: 78/66%, respectively) (Table 2).

## Indications for Cellular Therapies Today and Outlook

Traditionally, indications for HSCT in CGD had been the following: (1) > 1 invasive life-threatening infections, (2) non-tolerability of prophylactic drugs, (3) non-compliance, (4) severe autoinflammation, or (5) unavailability of a CGD-experienced physician (14, 21, 22, 111). Due to the above mentioned favorable results, there is nowadays agreement that HLA-matched HSCT is indicated in any CGD-patient with absent NADPH-oxidase enzyme activity (16). Small children with CGD may clearly benefit from 5/6- or 6/6-HLA-matched CBT in experienced centers. Less than 10/10-HLA-MUD should probably not be offered to asymptomatic CGD-patients since the rates of graft failure and chronic GVHD are higher than in completely matched transplants. The indication for HSCT in adults should be carefully assessed by the treating physician, although the results in recent years have been encouraging (38, 63, 67, 78, 84, 112). We believe that haploidentical transplants and GT in X-CGD should only be offered to high-risk CGD-patients suffering from severe infectious and/or autoinflammatory complications with no other treatment alternatives. Ideally, high-risk CGD-patients without matched donors should be prospectively investigated in trials comparing GT vs. haploidentical HSCT.

## Author Contributions

All authors listed have made a substantial, direct and intellectual contribution to the work, and approved it for publication.

## Conflict of Interest

The authors declare that the research was conducted in the absence of any commercial or financial relationships that could be construed as a potential conflict of interest. The handling editor AL declared a collaboration with the authors RC, TG.

## Abbreviations

aGVHD: acute graft-vs. -host disease
AIHA: autoimmune hemolytic anemia
AR-CGD: autosomal recessive CGD
BM: bone marrow
Bu: busulfan
CGD: chronic granulomatous disease
cAUC: cumulative area under the curve
CBT: cord blood transplantation
cGVHD: chronic graft-vs.-host disease
CY: cyclophosphamide
DHR: Dihydrorhodamin test
DC: donor chimerism
DLI: donor lymphocyte infusion
EBMT: European Group For Bone and Marrow Transplantation
EFS: event-free survival
Flu: fludarabine
FU: follow-up
GBS: Guillain Barré syndrome
GT: gene therapy
GVHD: graft-vs.-host disease
HSC: hematopoietic stem cell
HSCT: hematopoietic stem cell transplantation
IFI: invasive fungal infection
IBD: inflammatory bowel disease
ITP: idiopathic thrombocytopenic purpura
MAC: myeloablative conditioning
MUD: matched unrelated donor
Haplo: haploidentical
Mel: melphalan
MMUD: mismatched unrelated donor
MSD: matched sibling donor
Ped: pediatric
NBT: Nitroblue tetrazolium test
OS: overall survival
PB: peripheral blood
PGLD: progressive granulomatous lung disease
PTLD: post-transplant lymphoproliferative disorder
RIC: reduced intensity conditioning
TBI: total body irradiation
TNI: total nodal irradiation
TDM: therapeutic drug monitoring
Treo: treosulfan
TRM: transplant-related mortality
TT: thiotepa
UCB: umbilical cord blood
X-CGD: X-linked CGD.
